# Case Report:Pregnancy and birth in a mild phenotype of Alström syndrome

**DOI:** 10.3389/fgene.2022.995947

**Published:** 2022-10-03

**Authors:** Luca Marozio, Francesca Dassie, Gianluca Bertschy, Emilie M. Canuto, Gabriella Milan, Stefano Cosma, Pietro Maffei, Chiara Benedetto

**Affiliations:** ^1^ Department of Obstetrics and Gynecology, University of Turin, Turin, Italy; ^2^ Department of Medicine, University of Padua, Padua, Italy

**Keywords:** hyperandrogenism, obesity, ciliopathies, pregnancy, Alström syndrome, delivery, preeclampsia

## Abstract

**Background:** Alström syndrome (AS) is an ultrarare multisystemic progressive disease caused by autosomal recessive variations of the *ALMS1* gene (2p13). AS is characterized by double sensory impairment, cardiomyopathy, childhood obesity, extreme insulin resistance, early nonalcoholic fatty liver disease, renal dysfunction, respiratory disease, endocrine and urologic disorders. In female AS patients, hyperandrogenism has been described but fertility issues and conception have not been investigated so far.

**Case:** This case report describes the spontaneous conception, pregnancy, and birth in a 27-year-old woman with AS, characterized by a mild phenotype with late onset of visual impairment, residual perception of light, and hypertension. Before pregnancy, menses were regular with increased levels of dihydrotestosterone and androstanediol glucuronide in the follicular phase, and the ovaries and endometrium were normal during vaginal ultrasound. A thorough clinical follow-up of the maternal and fetal conditions was carried out. A weight gain of 10 kg during pregnancy was recorded, and serial blood and urine tests were all within the normal range, except for mild anemia. The course of pregnancy was uneventful up to 34 weeks of gestation when preeclampsia developed with an abnormally high level of blood pressure and edema in the lower limbs. At 35 weeks + 3 days of gestation, an urgent cesarean section was performed, and a healthy male weighing 1,950 g was born. Histological examination of the placenta showed partial signs of flow obstruction, limited abruption areas, congested fetal vessels and villi, and a small single infarcted area.

**Conclusion:** The present case demonstrates for the first time that conceiving is possible for patients with ALMS. Particular attention should be given to the management of AS systemic comorbidities through the course of pregnancy.

## 1 Introduction

Alström syndrome (AS) is a progressive multisystemic ultrarare autosomal recessive genetic disorder due to different genetic alterations on the *ALMS1* gene in chromosome 2p13.1 (OMIM #203800, prevalence: 1–9 cases per million inhabitants). Mutational hotspots are in exons 8 (6.1 kb), 10 (1.9 kb), and 16 (1.2 kb), containing almost 90% of described pathogenetic variants. ALMS1 protein is widely expressed, and its function is still unknown; it is associated with the centrosome and the basal body of the primary cilium, which suggests that it could play a role in ciliogenesis and ciliary function. In literature, at least two different AS phenotypes have been described: typical and mild. The typical phenotype is characterized by cone–rod dystrophy with several visual impairments, deafness, metabolic comorbidities with early extreme insulin resistance (IR) and type 2 diabetes mellitus, nonalcoholic fatty liver disease, dilated cardiomyopathy, progressive renal and liver failure, hypothyroidism, female hyperandrogenism, and male hypogonadism. Signs and symptoms usually appear in early infancy with great variability in age of onset and severity. In literature, recently, a mild phenotype was described; it has been featured by the slow onset of visual impairment and photophobia with preserved or mildly impaired hearing function, the absence of hyperphagia and childhood obesity, and mild metabolic complications. There is no described correlation between the genotype and phenotype, but we have reported that patients with a mild phenotype carried at least one pathogenic variant with alteration localized upstream of exon 7, suggesting a possible link between disease severity and variations’ position (Marshall et al., 2015; Astuti et al., 2017; Tahani et al., 2020; Dassie et al., 2021a; Dassie et al., 2021b).

Regarding fertility issues, female AS patients were commonly affected by hyperandrogenism, and they could be clinically characterized by the presence of oligomenorrhea, precocious puberty, polycystic ovaries, endometriosis, hirsutism, and alopecia. Women might also present with abnormal breast development with normal external genitalia, uterus, and fallopian tubes. Moreover, hypothyroidism, obesity, and IR that are typical features of AS patients can make ovarian cysts worse. In literature, there are no data on the sexual development and function in patients with mild AS phenotype (Marshall et al., 2005; Han et al., 2018).

Despite normal genital organs and less sexual hormonal impairment in female AS patients as compared to male AS patients, no pregnancy has been reported worldwide, and experts have considered fertility in these patients to be unlikely. The present case report describes the fertility characteristics, first spontaneous conception, course of pregnancy, and delivery by a woman with genetically confirmed Alström syndrome characterized by a mild phenotype.

## 2 Case description

We report the case of a 27-year-old pregnant nulliparous woman with genetically confirmed Alström syndrome (Marshall et al., 2005; Dassie et al., 2021a). The patient carries a homozygous nonsense substitution in exon 5 of *ALMS1* genotype *ALMS1* c.[1046G>A]+[1046G>A] p.(Trp349*)+(Trp349*) (Marshall et al., 2005; Marshall et al., 2015); the patient’s sister was also affected by Alström syndrome, carrying the same pathogenic variants in the homozygote state, while both parents were heterozygous asymptomatic carriers. The patient’s pathogenic variants had been identified at the age of 21 years during a genetic screening of cases with various subtypes of retinal dystrophies by different next-generation sequencing (NGS) platforms (Nasser et al., 2018; Dassie et al., 2021a).

Both the case patient and her sister had a mild AS phenotype. The patient’s phenotype was characterized by the presence of nystagmus in infancy, late onset of visual impairment with a residual perception of light and dark, and hypertension treated with ACE inhibitors associated to mild cardiac fibrosis with preserved ejection fraction at cardiac magnetic resonance imaging (MRI). From a metabolic point of view, the patient had no history of hyperphagia; she had no childhood obesity; and her prepregnancy body mass index (BMI, weight in kilograms divided by height in meters squared) was 27. The prepregnancy weight was 59 kg, the glycemic profile was normal (fasting glucose 4.9 mmol/L, HBa1c 34 mmol/mol), and the oral glucose tolerance test (OGTT) performed when the patient was 22 years old showed normal glucose, insulin, and C-peptide levels (glucose 0’: 4.8 mmol/L, glucose 120’: 6.7 mmol/L; insulin 0’: 3, insulin peak at 120’: 64; C peptide 0’: 1.6 ug/L, C peptide 120’: 10.4 ug/L). The patient had no dyslipidemia or hepatic and nephrological impairment, and the auditory tests were normal before pregnancy.

From a gynecological point of view, menarche occurred at the age of 12 years, and the following menses were regular. She had a Tanner stage of 5 for breast development and 3 for pubic hair representation. In the year before her pregnancy, the hormonal evaluation during the follicular phase showed normal levels of FSH and LH, prolactin (HPRL), 17-beta-estradiol, progesterone, testosterone, dehydroepiandrosterone sulfate (DHEAS), sex hormone binding globulin (SHBG), TSH, insulin, and fasting glucose and mild elevation of dihydrotestosterone (DHT 1.33 nmol/L, normal values: 0.08–1.26) and androstanediol glucuronide (7.70 ug/L, normal values: 0.34–7.53). The biochemical and hormonal assessments are shown in Table 1. Vaginal ultrasound showed a normal ovarian density and proliferative phase endometrium.

**TABLE 1:** Biochemical, metabolic, and hormonal test results.

Blood Tests	Normal Value	Basal Evaluation	Prepregnancy	Second Trimester of Pregnancy	First Trimester of Pregnancy	Post Pregnancy
Hemoglobin (g/L)	123–153	144	136	132	134	138
Fasting glucose (mmol/L)		4.4	4.6	4.6	4.1	4.8
Glucose 120′ (mmol/L)		6.7				
Fasting insulin (mU/L)		3	3	9.2	2.4	8.4
Fasting C-peptide (ug/L)	0.80–4.20	1.6	1.5	2.0	1.5	2.26
HbA1c (mmol/mol)		34	35	34	35	36
Total cholesterol (mmol/L)	2.00–6.19	5.79	5.38	7.74	4.76	6.40
LDL cholesterol (mmol/L)	1.00–4.12	5.79	3.80	5.09	3.11	4.53
HDL (mmol/L)	0.80–3.0	1.80	1.81	2.48	1.63	1.43
Triglycerides (mmol/L)		0.73	0.59	1.97	0.83	0.93
Uric acid (mmol/L)	0.15–0.35	0.24	0.20	0.19	0.18	0.26
Creatinine (umol/L)	45–84	56	64	53	53	57
Microalbuminuria (mg/L)		39.4	14.5	7.2	34.0	83.7
Ammonium (umol/L)	11–35	35	20			
GOT (U/L)	7–35	43	32	16	24	122
GPT (U/L)	10–35	30	24	14	22	59
ALP (U/L)	33–98	59	53	82	63	106
ɣ-GT (U/L)	3–45	32	78	11	57	202
Alpha amylase (U/L)	13–53	34	31			31
Urinary protein (g/L)		0	0	0	0.15	<10
Testosterone (nmol/L)	0.52–2.43	1.95	2.21			
DHT (nmol/L)	0.08–1.26	1.10	1.39	0.96	1.04	1.36
Estradiol (pmol/L)		376	436	50,000	10,155	250
DHEAS (umol/L)	0.90–9.21	7.5	9.7	4.28	5.8	4.28
Progesterone (nmol/L)		3.99	1.410	164.600	62.040	1.6
FSH (U/L)		6.3	4.5	0.1	0.1	5.9
LH (U/L)		5.3	5.5	0.1	0.2	5.4
HPRL (ug/L)		24.4		162.9	90.2	
SHBG (nmol/L)	18.0–144	45.9	39.8		316	
Androstanediol glucuronide (ug/L)	0.34–7.53	12.20	15.30	1.70	3.20	6.30
Androstenedione (nmol/L)		14.70	14.10	13.0	12.5	8.3
Aldosterone (pmol/L)		952	780	920	1030	481
Cortisol (nmol/L)	185–624	452	196	560	494	350
FT4 (pmol/L)	9–22	12.49	11.42	8.33	10.80	11.34
TSH (U/L)		2.37	1.40	1.95		1.01

In June 2020, at the age of 26 years, the patient conceived spontaneously. ACE inhibitors were suspended, and she was supplemented with folic acid, vitamin D, and iron supplements.

The patient presented to the gynecological outpatient department of Turin University in September 2020 at 13 weeks of pregnancy. A genetic assessment was performed; her partner was not a healthy carrier of AS; and a recurrence risk less than 1/18,000 was estimated.

**FIGURE 1:**
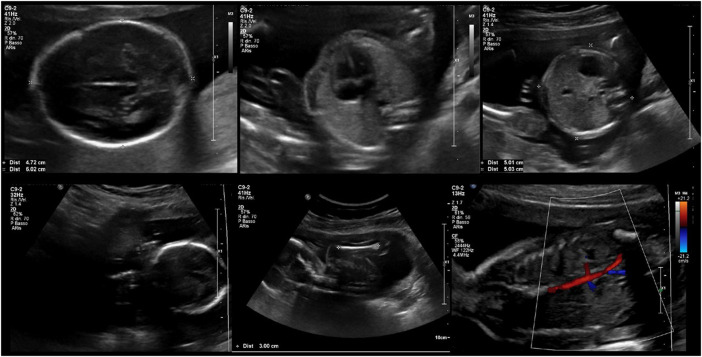
Fetal ultrasound assessment at 20 + 4 weeks of gestation.

A thorough clinical follow-up of maternal and fetal conditions was carried out. During pregnancy, a weight gain of 10 kg was recorded. Serial blood and urine tests (full blood count, coagulation profile, LDH, GOT/GPT, ɣ-GT, alkaline phosphatase, creatinine, haptoglobin, urinalysis, electrolytes, 24-hour proteinuria, and urine culture test) were all within the normal range, except for mild anemia. Screening for fetal chromosomal anomalies was negative: nuchal translucency of 1.6 mm; a risk minor than 1/100,000 for Down syndrome and a risk of 1/5,300 for neural tube defects were estimated. No fetal anomalies were detected at the second-trimester ultrasound screening. The ultrasound scans at 29 and 34 weeks of gestation disclosed normal fetal growth, anatomy, and Doppler flow of umbilical arteries (Figure 1). The trimestral maternal echocardiograms showed substantially stable parameters throughout pregnancy, with normal ejection fraction (57%), longitudinal deformation of the left ventricle, mild pericardial effusion (that was already present before pregnancy), and no signs of valvulopathy or pulmonary hypertension.

Regarding metabolic profile, OGTT at 20 weeks of gestation showed a normal level of glucose (fasting glucose) of 83 mg/dl (4.61 mmol/l), glucose after 60′ of 144 mg/dl (8 mmol/l), and glucose at 120’ of 110 mg/dl (6.11 mmol/l); HBa1c and insulin were also within the normal range throughout pregnancy (Table 1).

Due to a history of hypertension before pregnancy, the patient was instructed to measure her blood pressure at home regularly. She reported normal blood pressure values, which were confirmed at monthly visits for up to 33 weeks of gestation; no antihypertensive therapy was prescribed until then. However, she was hospitalized for abnormally high blood pressure and clinically evident edema of the lower limbs at 34 weeks of gestation. On admission, corticosteroid prophylaxis to prevent fetal respiratory distress syndrome (RDS) was administered. The laboratory tests showed elevated levels of 24-hour proteinuria, a nondiagnostic result for the ratio of soluble fms-like tyrosine kinase 1 (sFlt-1) to placental growth factor (PlGF: a marker that can be used to rule out short-term preeclampsia in women in whom the syndrome is suspected clinically), and bile acids levels above the normal range (Zeisler et al., 2016). Pharmacological treatment with nifedipine 20 mg and ursodeoxycholic acid 450 mg twice daily was initiated. Owing to poor treatment response, antihypertensive therapy was switched to alpha methyldopa (500 mg thrice a day) and labetalol (100 mg once a day), which yielded better results though the blood pressure remained elevated.

Cardiotocography and Doppler flow ultrasound monitoring of umbilical arteries were performed twice and once a day, respectively. The previous diagnostic tests were within the normal range, as were the fetal growth curves. In addition, noninvasive maternal hemodynamic assessment using an ultrasound cardiac output monitor (USCOM®) revealed a vasoconstricted and hypo dynamic profile characterized by elevated peripheral resistance, low stroke volume, and low cardiac output.

Considering the patient’s preeclampsia and elevated blood pressure, a cesarean section was performed at 35 weeks + 3 days of gestation. A healthy male weighing 1,950 g was born (Apgar score of 9 at 1 and 5 min). The histological examination of the placenta (weight, 350 g) showed partial signs of flow obstruction, limited abruption areas, congested fetal vessels and villi, and a small single infarcted area.

The patient and her baby were discharged 6 days after the cesarean section. No breastfeeding was performed after the delivery; the patient restarted ACE inhibitors; and the newborn had a regular growth. The patient was evaluated 6 months after the delivery, and she had normal blood pressure values with unmodified data at echocardiography analysis; biochemical, metabolic, and hormonal assessments showed increased levels of GOT, GPT, and ɣ-GT with normal glycemic profile. At 6 months of age, the newborn had no clinical abnormalities or any signs of retinal disease.

## 3 Discussion

To the best of our knowledge, this is the first case report to describe pregnancy at term in a woman with AS, a rare multisystemic genetic disorder that usually has a negative effect on both male and female fertility.

Women with AS clinically present with IR, hyperandrogenism, irregular menses, and chronic anovulation similar to the polycystic ovary syndrome (PCOS) phenotype. A recent analysis of endocrinological manifestations in the American cohort of female AS patients has confirmed increased testosterone levels when compared to controls (Han et al., 2018). Extensive ovarian fibrosis associated with poor follicular reserve has also been reported in postmortem analysis in patients with AS (Marshall et al., 2005). Regarding fertility and possible pregnancy, the 2020 Consensus Clinical Management Guidelines for AS states that “Female fertility is unlikely and no patients have reproduced so far” (Tahani et al., 2020).

The described patient shows a mild phenotype, and from a gynecological point of view, she differs from the typical phenotype because she has normal hair, normal breast conformation, and normal internal/external genitalia (Table 2); moreover, she has a normal metabolic profile that may have contributed to her fertility. In the literature, no data are available on fertility in female AS patients, and only one study in mice has suggested that female Alms1 foz/foz mice were fertile at an early age and became infertile after the development of obesity due to an anovulatory state (Arsov et al., 2006). The mild phenotype of this AS patient without IR or obesity, associated only with a mild initial increase of androgen levels, may have spared the patient's fertility and led to the possibility of a spontaneous conception. In fact, it is well known from PCOS studies that IR and hyperinsulinemia influenced fertility and conception, enhancing androstenedione and testosterone production, reducing SHBG levels, increasing free testosterone levels, stopping follicles growth, influencing pituitary gland sensitivity to GnRH, and stimulating pituitary LH release (Shaaban et al., 2021). A recent study in mice suggests a direct role of ALMS1 on fertility. This study showed that ALMS1 deficiency may promote anovulatory infertility *via* elevated androgens through a cooperation between ALMS1 and the luteinizing hormone (LH)/chorionic gonadotropin receptor, which induced a PCOS and obesity phenotype characterized by anovulation and hyperandrogenemia (Yu et al., 2021). As in the general population, female AS patients affected by obesity, metabolic syndrome, and their comorbidities may have ovulatory and anovulatory cycles, decreased oocyte number and quality, increased rate of miscarriages, and lower rates of pregnancy after medically assisted reproduction. In the literature, there are no data on this complex interaction in AS, but it is clear that a strict control of metabolic manifestations may ameliorate the fertility rate of female AS patients.

**TABLE 2:** Typical and mild gynecological phenotype of Alström syndrome.

	Typical Phenotype	Mild Phenotype
Hair	Alopecia and hirsutism	Normal
Breast	Abnormal breast development	Normal
Genitalia	Ovary cysts	Normal
Menarche and menses	A/Oligomenorrhea	Normal
Hormonal assessment	Hyperandrogenism	Mild increase in testosterone levels

Other AS characteristics not directly involved with the female reproductive system, such as hypothyroidism and GH deficiency, may further affect patients’ fertility. In fact, in a similar way, hypothyroidism and GH deficiency impact the morphology of the reproductive organs, the onset of puberty, ovarian function, and fertility and have to be considered to ameliorate fertility in patients with AS.

Regarding conception, considering the AS part of genetically determined ciliopathies, ALMS1 dysfunction may be involved in embryo implantation and pregnancy development. In fact, this case suggests that, as observed in other ciliopathies, male individuals are infertile (in the literature, there are no reports of a newborn from male patients affected by AS), while female AS patients may be fertile or infertile based on the functionality of motile cilia, which play a critical role in genitalia development, reproductive system, and hormonal function (Raidt et al., 2015). Nonmotile (primary) cilia dysfunction usually causes hypogonadism and genital abnormalities. Every primary ciliopathy is associated with multiple pathogenetic variants and phenotypes, and these different genotypes and phenotypes affect both male and female fertility in different ways; a brief summary of the fertility issues in main syndromic primary ciliopathies is reported in Table 3. Actually, it is not known whether spontaneous miscarriages or termination of pregnancy occur in AS patients with mild and typical phenotypes, but the present case demonstrates that, although difficult, conceiving is not impossible in these patients.

**TABLE 3:** Main primary ciliopathies and fertility issues.

	Male Fertility	Female Fertility	Offspring	References
Polycystic kidney disease	Common male infertility with cysts in the epididymis, seminal vesicle, and ejaculatory duct	Female fertility preserved with possible cysts in the reproductive system	Described	Kanagarajah et al. (2012)
Nephronophthisis as part of multisystemic primary ciliopathies	Depends on associated phenotype	Depends on associated phenotype	Described	Devi et al. (2015); Yzer et al. (2012); Tezerjani et al. (2016)
Oral–facial–digital syndrome		X-linked type: miscarriage of male fetus and fertility preserved in female mild phenotype	Described	Iijima et al. (2019)
Bardet–Biedl syndrome	Common male infertility with male hypogonadism, genitourinary malformations (i.e., micropenis), and cryptorchidism	Common female infertility with hypogonadism and Possible anatomic defects such as hypoplastic or duplex uterus, hypoplastic fallopian tubes and/or ovaries, septate vagina, partial or complete vaginal atresia, absent vaginal and/or urethral orifice, hydrocolpos or hydrometrocolpos, persistent urogenital sinus, and vescicovaginal fistula	Described	Forsyth et al. (2003)
Alström syndrome	Common male infertility with male hypogonadism and genitourinary malformations (i.e., micropenis, hypospadias, and small testis)	Female fertility in typical phenotype characterized by hyperandrogenism, ovary cysts, and menses abnormalities

This case also highlights the importance of the management of pregnancy and delivery comorbidities in patients with AS. In fact, AS is characterized by an array of systemic clinical manifestations that may increase the risk of pregnancy and delivery complications, as demonstrated by this case that developed preeclampsia at 34 weeks of gestation. In fact, the patient was affected by chronic arterial hypertension with mild cardiac involvement, which are well-known risk factors for preeclampsia and fetal death (Ritter et al., 2020). Furthermore, in AS patients, the presence of IR, type 2 diabetes mellitus, nonalcoholic fatty liver disease and renal impairment are among the most frequent clinical manifestations of this syndrome and increase the risk for preeclampsia and eclampsia development (Raidt et al., 2015; Baig et al., 2020; Ritter et al., 2020; Bettini et al., 2021; Shaaban et al., 2021; Yu et al., 2021; Lee and Kim, 2022). What remains unclear is whether the obstetrical complications observed in our patient are directly related to genetic alterations. Studies on ciliopathies suggest a potential role of primary cilium dysfunction in eclampsia. The primary cilium is required for the functionality of human chorionic villi mesenchymal stromal cells, and the primary cilia are also impaired on human chorionic villi mesenchymal stromal cells from preeclamptic placentas (Romberg et al., 2022).

Another open question is how pregnancy influences the patients’ metabolism after pregnancy. We observed a worsening of hepatic function that seems unrelated to preeclampsia events. Actually, there are no clinical data on the follow-up of AS patients after pregnancy, but we suggest a careful metabolic and cardiovascular comorbidities assessment.

Finally, the clinical description of the AS phenotype suggests an impaired breast development that together with endocrinological impairment may compromise breastfeeding. In our case, the patient did not perform lactation because she never developed transition and mature milk. Breastfeeding impairment could be secondary to two mechanisms: (a) ALMS1 dysfunction could be directly correlated to milk production in glandular breast cells, and (b) as suggested in some observational studies, a PCOS-like phenotype associated to obesity could be a potential factor contributing to lower breastfeeding initiation and duration (Harrison et al., 2016; Author Anonymous, 1161).

From the patients' and Alström syndrome community perspective, this case report highlights that spontaneous conception and pregnancy are possible in female AS patients, and it suggests that strict control of metabolic and cardiovascular complications in childbearing age might improve fertility.

In conclusion, one particular issue of ciliopathies is fertility. In Alström syndrome, fertility can be preserved in female AS patients with a mild phenotype. Cardiovascular and metabolic comorbidities, even if absent or mild before conception, may be worsened and should be considered during pregnancy follow-up and delivery management. The spontaneous conception and pregnancy in this case report, for the first time, highlights the importance of counselling in women affected by AS, and they suggest that gynecologists may occasionally enter the multidisciplinary team for care of patients with AS.

## Data Availability

The data sets for this article are not publicly available due to concerns regarding participant/patient anonymity. Requests to access the data sets should be directed to the corresponding author.
